# Development of a New 3D Hybrid Model for Epithelia Morphogenesis

**DOI:** 10.3389/fbioe.2020.00405

**Published:** 2020-05-05

**Authors:** Filippos Ioannou, Malik A. Dawi, Robert J. Tetley, Yanlan Mao, José J. Muñoz

**Affiliations:** ^1^Institute for the Physics of Living Systems, University College London, London, United Kingdom; ^2^MRC Laboratory for Molecular Cell Biology, University College London, London, United Kingdom; ^3^Laboratori de Càlcul Numèric (LaCàN), Universitat Politècnica de Catalunya, Barcelona–Tech, Barcelona, Spain; ^4^College of Information and Control, Nanjing University of Information Science and Technology, Nanjing, China

**Keywords:** wound healing, vertex, modeling, three dimensions, morphogenesis

## Abstract

Many epithelial developmental processes like cell migration and spreading, cell sorting, or T1 transitions can be described as planar deformations. As such, they can be studied using two-dimensional tools and vertex models that can properly predict collective dynamics. However, many other epithelial shape changes are characterized by out-of-plane mechanics and three-dimensional effects, such as bending, cell extrusion, delamination, or invagination. Furthermore, during planar cell dynamics or tissue repair in monolayers, spatial intercalation between the apical and basal sides has even been detected. Motivated by this lack of symmetry with respect to the midsurface, we here present a 3D hybrid model that allows us to model differential contractility at the apical, basal or lateral sides. We use the model to study the effects on wound closure of solely apical or lateral contractile contributions and show that an apical purse-string can be sufficient for full closure when it is accompanied by volume preservation.

## 1. Introduction

Many morphogenetic events in epithelia can be successfully described with two-dimensional models. Some examples include tissue intercalation (Munjal et al., 2015), jamming transitions (Bi et al., 2016), or collective cell migration (Sunyer et al., 2016). However, monolayers are also subjected to observable out of plane deformations like tissue folding (Tozluoğlu et al., 2019), invagination (Bielmeier et al., 2016), extrusion (Deforet et al., 2014), or delamination (Eisenhoffer et al., 2012). In these cases, two-dimensional models with folding capabilities (Misra et al., 2016), or purely three-dimensional models, usually implemented in the continuum context, seem necessary to capture the underlying contractile mechanisms.

There are also other problems that, despite being studied extensively in two-dimensions, contain three-dimensional contributions that have not been included in their modeling and description. Some examples are neural crest zippering (Hashimoto et al., 2015) or wound healing in monolayers (Antunes et al., 2013; Brugués et al., 2014). The former in fact originates from a precedent tissue folding, while experimental observations of the latter indicate that wound closure has some variations along the cell apicobasal axis (Zulueta-Coarasa et al., 2014). Understanding forces in these processes requires models that are able to reproduce both junctional mechanics and deformations that are different at the apical and basal sides of the monolayer. The present paper introduces a vertex model that includes these ingredients.

The discrete nature of tissues makes vertex modeling an ideal approach that has been successfully employed to simulate cell dynamics of monolayers and study, for example, T1 transitions (Bi et al., 2015), phase transformations (Bi et al., 2016), and wound healing (Brugués et al., 2014; Staddon et al., 2018; Tetley et al., 2019). For further reference on vertex models see for instance the recent review (Alt et al., 2017).

Three-dimensional versions are more scarce, but have been also recently developed to study curved monolayers (Gómez-Gálvez et al., 2018), cyst formation in monolayers (Bielmeier et al., 2016), folding in epithelial shells (Misra et al., 2016), or general morphogenesis (Okuda et al., 2013, 2015).

We here extend a previous version of our 2D hybrid model to three dimensions (Mosaffa et al., 2018), with the ability to uncouple intercalation on the apical and basal sides of the monolayer. Differential intercalation and the geometrical definition of the cell poses special computational challenges. The analysis of shape transitions between the two sides of a monolayer have been analyzed for curved monolayers (Gómez-Gálvez et al., 2018). Differential apical and basal intercalation has also been seen in the *Drosophila* salivary gland–termed “interleaving” (Sánchez-Corrales et al., 2018). A similar idea is employed here in flat geometries by defining an intermediate vertex that facilitates neighbors changes.

In our model, we resort to a hybrid approach, where the cell-centers of the apical and basal layer are the main degrees of freedom. As is customary in vertex models, each cell-center is surrounded by a set of vertices, where mechanical balance is also imposed between all connected bar elements (Barton et al., 2017). Vertex positions are constrained by the triangulation of the cell-centers. By inserting these constraints into the equilibrium equations, we manage to reduce the computational size of the model, which is solely described by cell-center positions, and thus simplifies the topological definition of the monolayer. We note though that despite this reduction, our final equations still include vertex equilibrium.

Illustrative results are shown by applying the model for the analysis of wound healing, with specific contractility evolution at the wound edge. Each bar element of the model adopts a viscoelastic rheology, which is based on a dynamic change of the rest-length (Muñoz and Albo, 2013; Staddon et al., 2018), and allows calibrating the short term recoil process.

## 2. Methodology

### 2.1. Three-Dimensional Vertex Model

#### 2.1.1. Monolayer Geometry

The cells in the monolayer are initially defined by the centers (*nodes*) of their surfaces at the apical (top) and basal (bottom) sides, respectively denoted by $x_{A}^{i}$ and $x_{B}^{i}$, *i* = 1, …, *N*_*cells*_. In our simulations, these cell centers are equal at the apical and basal sides, and correspond to the measured experimental locations of the cell center of mass.

**Figure 1:**
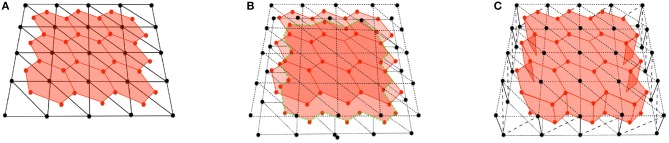
**(A)** Scheme of a cell with vertex bar elements (red) and nodal bar elements (black). **(B)** Adding apical vertex (red) and nodal (black) network. **(C)** Construction of cells with vertical bar elements. Diagonal elements in vertex network have been omitted for clarity.

After applying a 2D Delaunay triangulation on each side, the cell apical, and basal boundaries are then constructed by joining the *vertices* $y^{I},I=1,\ldots ,N_{y}$ *on each side. These vertices are located at the barycenters of the triangles that surround each cell center*. The two 2D layers are then joined with vertical and diagonal segments that join apical and basal nodes, and also basal and apical vertices, forming prism-like polyhedra. As a result, each cell is formed by an apical and basal center (node) and two sets of vertices defining the apical and basal boundaries. Figure 1 shows the construction process and final polyhedra.

**Figure 2:**
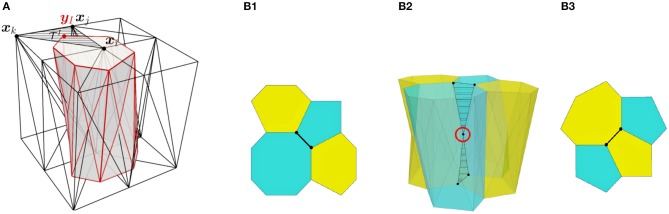
**(A)** Scheme of a cell with vertex bar elements (red) and nodal bar elements (black). **(B)** Definition of intermediate vertices in order to ease different connectivity at basal surface **(B1)** and apical surface **(B3)**.

The initial position of the apical and basal cell-centers (nodes) are taken from two dimensional experimental images, where cell center positions are measured. The cell boundaries are located at the barycenters of the resulting triangles computed from a Delaunay triangulation of the cell centers. Consequently, the initial cell-center locations and shape of the cell areas at the apical and basal surfaces are equal. However, they are allowed to change their connectivity independently in subsequent time-steps, forming polyhedra that may have different polygonal shapes and number of sides at each apical and basal surface. The apico-basal transition between two polygons with different number of segments is facilitated by the definition of *intermediate vertices*, located between the apical and basal surfaces, as shown in Figure 2.

More specifically, the positions of the vertices at the apical and basal surfaces are computed from the interpolation of the corresponding cell-centers by using the following constraint equation,

(1) $$\begin{array}{l} y^{I}=\overset{3}{\underset{i=1}{\sum }}N^{i}\left(\xi^{I}\right)x_{I}^{i}s,I=1,\ldots ,N_{tri} \end{array}$$

where *N*^*i*^(**ξ**^*I*^) are interpolation functions associated to each one of the nodes $x_{I}^{1},x_{I}^{2},x_{I}^{3}$ forming triangle *I*, and **ξ**^*i*^ a parametric coordinate. Hereafter we set *N*^*i*^(**ξ**^*I*^) = 1/3, so that vertices are located at the triangle barycenters. As a consequence, the model is a hybrid version between a cell-centered and a purely vertex model. Vertices are used for defining the cell boundary (cortex), but cell-centers are kept as degrees of freedom (DOF) describing the whole cell kinematics. Similar approaches can be found in Barton et al. (2017) and Mosaffa et al. (2018).

The resulting geometrical construction is formed by two coupled networks: the set of segments *ij* joining nodes *nodal network*, and the segments *IJ* joining the vertices *vertex network*. The motivation of the hybrid approach is threefold: (i) it reduces the number of DOF, and thus the size of the resulting system of equations to solve at each time-step, (ii) it allows us to model mechanical interactions between cell-centers (nodal network of triangles) and joint mechanics (vertex network of polygonal cell boundaries), and (iii) it also provides a mechanical coupling between the two networks.

In order to define the vertices at the boundary of the patch, we add a set of boundary nodes which are not the center of any cell. We note also that intermediate vertices are free to move as independent DOF, so that they are not interpolated according to Equation (1). Furthermore, we will also relax the constraint in Equation (1) for those vertices that are at the edge of the wound, and let all those free vertices, denoted by $y_{w}^{I}$, to be additional DOFs. As a result, the kinematics of the monolayer is fully defined by the cell centers positions ***x***^*i*^ and the coordinates of the relaxed vertices $y_{w}^{I}$. We note that this relaxation is introduced in order to avoid zig-zag effects at the wound edge. The reader is referred to Mosaffa et al. (2018) for further analysis on this effect.

#### 2.1.2. Mechanical Equilibrium

In each one of the nodal and vertex network, we distinguish apical, basal and lateral segments. The total energy of the system *W*(***x***, ***y***_*w*_) is defined by the sum of nodal and vertex elastic contributions, and a volume penalization as,

(2) $$\begin{array}{l} W\left(x,y_{w}\right)=W_{N}\left(x\right)+W_{V}\left(y\left(x\right),y_{w}\right)+W_{Vol}\left(y\left(x\right),y_{w}\right) \end{array}$$

Each term is defined by,

$$\begin{array}{l} \;W_{N}(x)=\frac{k_{N}}{2}\sum_{ij}(l^{ij}-L^{ij}{)}^{2}\\ \;W_{V}\left(y(x),y_{w}\right)=\frac{k_{V}}{2}\sum_{IJ}{\left(l^{IJ}-L^{IJ}\right)}^{2}\\ W_{Vol}\left(y(x),y_{w}\right)=\frac{\lambda_{Vol}}{2}\sum_{i=1}^{N_{cells}}{\left(\frac{V^{i}-V_{0}^{i}}{Vi_{0}}\right)}^{2} \end{array}$$

The material parameters *k*_*N*_ and *k*_*V*_ measure the stiffness of the nodal and vertex segments, respectively. The measures *l*^*ij*^ = ||***x***^*i*^ − ***x***^*j*^|| and *l*^*IJ*^ = ||***y***^*I*^ − ***y***^*J*^|| correspond to the current observable lengths of each nodal and vertex segment. The rest-lengths *L*^*ij*^ and *L*^*IJ*^ are internal variables, not necessarily constant. Their evolution will furnish viscous properties to the monolayer, and will be defined in section 2.1.3. The penalization parameter λ_*Vol*_ is set to a value such that relative volume difference $|V^{i}-V_{0}^{i}|/V_{0}^{i}$ at each cell *i* is kept between 5 and 10%, as experimentally measured (Gelbart et al., 2012).

Mechanical equilibrium is achieved by minimizing the total energy with respect to the positions of the nodes and the relaxed vertices, i.e.,

(3) $$\begin{array}{l} \frac{\partial W_{N}}{\partial x^{i}}+\frac{W_{V}}{\partial y^{I}}\frac{\partial y^{I}}{\partial x^{i}}+\frac{\partial W_{Vol}}{\partial y^{I}}\frac{\partial y^{I}}{\partial x^{i}}=0,\;i=1,\ldots ,N_{nodes} \end{array}$$

(4) $$\begin{array}{l} \frac{W_{V}}{\partial y_{w}^{I}}+\frac{\partial W_{Vol}}{\partial y_{w}^{I}}=0,I=1,\ldots ,N_{relax} \end{array}$$

The terms $\frac{\partial y^{I}}{\partial x^{i}}$ are computed from the constraint equation in (1) as, $\frac{\partial y^{I}}{\partial x^{i}}=N^{i}\left(\xi^{I}\right)\text{I}=1/3\text{I}$, with $\text{I}=\frac{\partial x^{i}}{\partial x^{i}}$ the three-dimensional identity matrix. Due to the non-linearity of the equations in (3), the solution is found using a Newton-Raphson strategy enhanced with line-search strategies.

After each converged step at time *t*_*n*_, with connectivity *C*_*n*_ and nodal and vertex positions $x_{n}^{i}$ and $y_{w}^{I}$, we compute new nodal and vertex positions, $x_{n+1}^{i}$ and $y_{w,n+1}^{I}$, respectively, and a new connectivity *C*_*n*+1_ using a modified Delaunay triangulation of the apical and basal surfaces. New triangles are formed if their aspect ratio at time *t*_*n*+1_, denoted by *r*_*n*+1_, is improved according to the relation

$$r_{n+1}<\left(1+tol_{r}\right)r_{n}$$

with *tol*_*r*_ a non-negative numerical parameter. When *tol*_*r*_ = 0, the standard Delaunay algorithm is recovered, while for *tol*_*r*_ > 0, suboptimal stretched triangles and cells are permitted.

#### 2.1.3. Rheological Model

Each vertex segment in the model has the ability to respond according to a viscoelastic rheological law. This law is implemented by resorting to a variable rest-length *L*^*ij*^ or *L*^*IJ*^, which evolves with the following equation:

(5) $$\begin{array}{l} \frac{1}{L}\frac{dL}{dt}=\gamma \left(\frac{l-L}{L}-\varepsilon^{c}\right) \end{array}$$

This law reflects the fact that as far as the strain measure (*l* − *L*)/*L* is different from a contractility ε^*c*^, the rest-length will evolve, and that *L* will remain unchanged when the strain reaches the value ε^*c*^. It has been proved that for ε^*c*^ = 0, such evolution law gives a similar response to a Maxwell viscous model (Muñoz and Albo, 2013). The intrinsic contractility ε^*c*^ has been included in order to mimic the contractile state of cells (Khalilgharibi et al., 2019; Wyatt et al., 2020).

In addition, we also consider the local actin concentration at the wound edge. This is implemented through an additional contractility ${\hat{\Upsilon }}^{c}$, which increases the tension at the elastic branch of the vertex segment as

$$\sigma_{V}=k_{V0}\left(\varepsilon^{e}+\Upsilon^{c}\right)$$

We will use time-varying values of ${\hat{\Upsilon }}^{c}$ at the apical and lateral sides of the cells, as it is explained in section 2.2. As a result, an additional tension equal to $k_{V0}{\hat{\Upsilon }}^{c}$ is being applied on those vertex segments.

**Figure 3:**
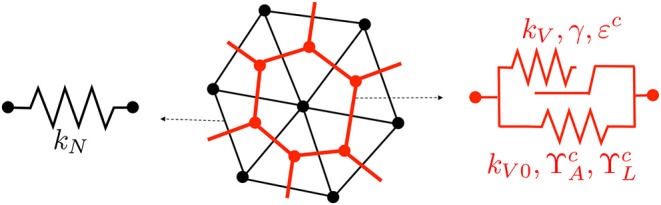
Rheological model for nodal **(left)** and vertex **(right)** segments. Contractility parameters $\Upsilon_{A}^{c}$ and $\Upsilon_{L}^{c}$ are only used at the wound edge.

Figure 3 depicts the rheological model employed at the nodal and vertex segment. The purely elastic behavior is obtained by the minimization of energies *W*_*N*_ and *W*_*V*_ using, respectively, stiffnesses *k*_*N*_ and *k*_*V*0_, while the viscous response results from the implementation of the evolution law in (5) in an additional energy term *W*_*V*_ with stiffness *k*_*V*_. For simplicity and to avoid having to fit too many parameters, we have set γ = 0 for the nodal elements, which yields a purely elastic behavior, as depicted in Figure 3. The calibration of the material parameters will be explained in section 3.1.

#### 2.1.4. Numerical Solution

The set of non-linear equations in (3) depends non-linearly on the nodal positions ***x***^*i*^ and on the relaxed vertices $y_{w}^{I}$. In addition, these equations, which may be expressed as,

(6) $$\begin{array}{l} g\left(x,y_{w}\right)=0 \end{array}$$

also include the rest-lengths *L* of each bar element. These rest-lengths obey the differential equation in (5), which is discretized in time using a θ − *weighted* scheme,

(7) $$\begin{array}{l} L_{n+1}-Ln=\Delta tL_{n+\theta }\gamma \left(\frac{l_{n+\theta }-L_{n+\theta }}{L_{n+\theta }}-\varepsilon^{c}\right) \end{array}$$

with (•)_*n*+θ_ = (1 − θ)(•)_*n*_ + θ(•)_*n*+1_. We used the value θ = 0.5, which yields a second-order accurate and unconditionally stable scheme in linear systems. The relation in (7) allows obtaining an expression of *L*_*n*+1_ as a function of *l*_*n*+1_ and other values at time *t*_*n*_. This expression is inserted in the system of equations in (6) at each time *t*_*n*+1_, and solved in an implicit manner with a fully Newton-Raphon iterative process. We set the convergence tolerance *tol* = 1*E*−10 and impose the convergence condition ||δ***x***|| < *tol* and ||***g***(***x***, ***y***_*w*_)|| < *tol*. We have used the time-step size Δ*t*_1_ = 0.6 min during the recoil process (up to *t* = 6 min), and Δ*t*_2_ = 1 min during the closure process. In some simulations, the latter time-step was halved in some of the increment in order to achieve convergence. The total simulation when using a patch of 205 cells took around 150 increments, and a run time of approximately 20 min in Matlab R2018a in a Windows machine with Intel(R) Core(TM) i7-6700 CPU @ 3.4 GHz, and 16 GB RAM memory, working with 2 processors.

### 2.2. Wounding and Contractility

The *Drosophila* larval wing imaginal disc is a pseudostratified epithelium containing highly columnar cells. This tissue is therefore an ideal experimental system for investigating wound healing cell behaviors in 3-dimensions. We wounded wing disc epithelia by ablating multiple tricellular junctions at the level of adherens junctions using a pulsed TiSa laser (Tetley et al., 2019). We then imaged the wound healing response in wing discs expressing a GFP tagged form of non-muscle Myosin II using 3D time-lapse confocal microscopy with intervals of 3 min between successive time points. A representative sequence of images is shown in Figure 5.

We simulate the *in silico* wounding of the vertex model by degrading the stiffness of the ablated cells down to 1% of their initial value, and removing the volume penalization term in the total energy for these cells, that is, reducing λ_*Vol*_ to 0 on those ablated cells. This softening, together with the subsequent removal of these degraded cells and the progressive intercalation of the cells at the wound edge allows us to simulate progressive wound closure.

**Figure 4:**
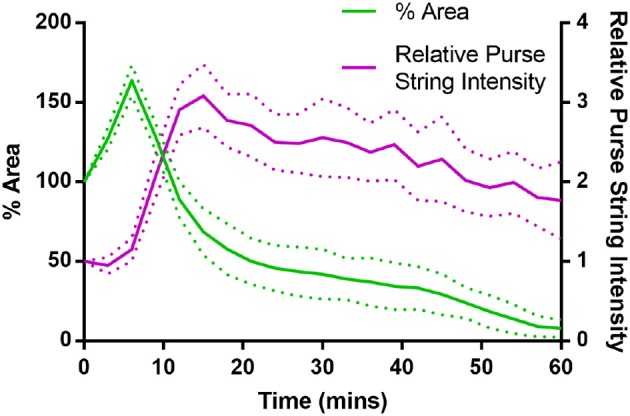
Purse-string Myosin II intensity and wound area evolution during the first hour of wound closure, averaged over 5 wing disc wounds. Myosin II intensity in the purse-string gradually reduces over time, as the wound closes (Error bars = S.D.).

We explicitly implement the evolution of an actomyosin purse-string at the wound edge by applying a decreasing trend to the apical vertex segments, while applying a constant value at the lateral sides. In the model, the contractilities are explicitly given by,

$$\begin{array}{l} {\hat{\Upsilon }}_{A}^{c}=\{\begin{array}{ll} \Upsilon_{A}^{c}\left(1-\frac{t-t_{w}}{400}\right), & t>t_{w}\\ 0, & \text{otherwise} \end{array}\\ {\hat{\Upsilon }}_{L}^{c}=\{\begin{array}{ll} \Upsilon_{L}^{c}, & t>t_{w}\\ 0, & \text{otherwise} \end{array} \end{array}$$

where *t*_*w*_ ≈ 6 min is the time at which purse-string contractility is activated, with *t* = 0 the time for tissue ablation. Our simulations last in average around 150 min, so that the factor 1/400 in ${\hat{\Upsilon }}_{A}^{c}$ aims at reducing the purse-string contractility with a similar trend of the concentration of Myosin II measured and showed in Figure 4. Model parameters $\Upsilon_{A}^{c}$ and $\Upsilon_{L}^{c}$ will be calibrated in section 3.1.

**Figure 5:**
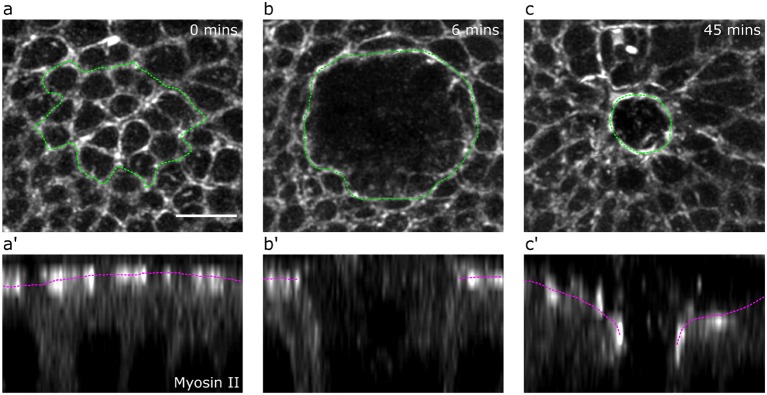
*Drosophila* wing disc wound evolution. Apical **(a,b,c)** and lateral views **(a’,b’,c’)** of a *Drosophila* wing disc expressing a GFP-tagged form of non-muscle Myosin II at 0 min **(a,a’)**, 6 min (maximum recoil, **b,b’**) and 45 min (partial closure, **c,c’**) after wounding. The margin of killed cells **(a)** and the wound **(b,c)** are marked by a green dotted line. The apical surface of the tissue is marked by a magenta dotted line **(a’,b’,c’)**. Scale bar = 5μm.

### 2.3. Experimental Measurements

Experimental quantifications were averaged across wounds in five separate wing discs. We quantified the evolution of wing disc apical wound area by manually tracing the periphery of the wound for the first 72 min after wounding. The wound periphery was particularly clear while wound healing progressed, due to the formation of an apical actomyosin purse-string (Figure 5). To quantify the evolution of wound apical indentation depth, we first generated orthogonal image views and fitted a line between the highest points of the apical surface either side of the wound edge. We then calculated the distance along the apicobasal axis between this line and the position of the wound periphery (most clearly marked by the actomyosin purse-string, Figure 5). The relative height was computed from the depth measurements by assuming that the monolayer had an average height of 35 μm.

**Figure 6:**
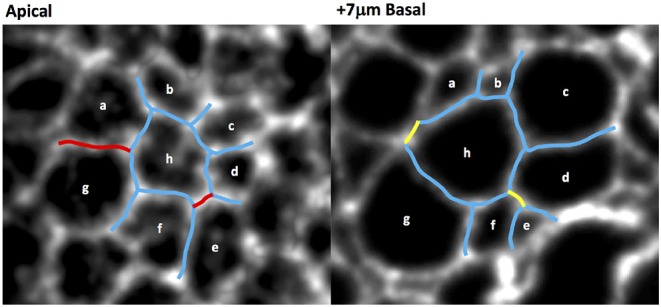
Wing disc cell outlines (cells a–h, marked by CAAX-GFP) at two different apicobasal positions, demonstrating that neighbor exchanges occur along the apicobasal axis at a single time point. Persistent cell-cell junctions are in blue, lost junctions in red and gained junctions in yellow.

We also quantified the number of T1 transitions by analysing the gradual reduction in the number of cells at the wound edge. The evolution of the latter will be compared with our simulations in the next section. Figure 6 demonstrates that T1 transitions also occur spatially, along the apicobasal axis in a single timeframe, as well as temporally in the plane of the epithelium. This justifies our inclusion of intermediate vertices (Figure 2).

## 3. Results

### 3.1. Model Calibration

**Figure 7:**
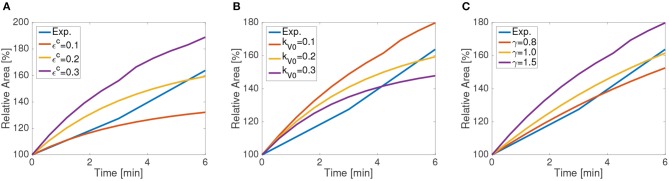
Apical wounded area as a function of time for the first 6 min following ablation as simulated using a 65 cell computational tissue. Different values of **(A)** background contractility ε^*c*^, **(B)** vertex stiffness *k*_*V*_, and **(C)** and remodeling rates γ.

We use the recoil process for calibrating tissue contractility ε^*c*^, vertex stiffness *k*_*V*0_, and the remodeling rate γ, which measures the viscous response. In order to avoid stiffness redundancy between vertex and nodal networks, we fix *k*_*N*_ = 0.5. Figure 7 shows the sensitivity of the recoil to these material parameters.

Tissue contractility ε^*c*^ increases the asymptotic line tension in cells, and as such increases the final apical and basal areas after recoil. Larger values of recoil for MyoII activation and higher tension have been reported experimentally (Tetley et al., 2019), supporting this effect. Material stiffness has an inverse trend, reducing recoil for larger values of *k*_*V*0_, which increases the relative energy cost of length changes. In our formulation, mechanical equilibrium is reached through energy minimization, which is proportional to material stiffness and line strains. Consequently, when stiffness increases, line stretching is in general reduced.

The remodeling rate mimics the viscous and fluid response of the cells. For higher values of γ, the characteristic time and the viscosity of the fluid is reduced, in agreement with the model (Muñoz and Albo, 2013). Consequently, the area after recoil increases for a fixed time. The final values of the tissue are given in Table 1. Other values of nodal and vertex stiffness, *k*_*N*_ and *k*_*V*_ have been manually fitted so that the final wound area remains stable at the experimental values, and that no element is under compression. Although there is some redundancy on their values (multiple combinations giving similar wound area), we have chosen the values *k*_*N*_ = 0.3 and *k*_*V*_ = 1.0.

**Table 1:** Values of parameters fitted in recoil phase.

**Parameter**	**Value**
Contractility ε^*c*^	1.3
Vertex stiffness *k*_*V*0_	0.05
Remodeling rate γ	0.2

*Fixed values are k_N_ = 0.3 and k_V_ = 1*.

### 3.2. Wound Healing Simulations

We tested squared patches with different size, from 80 to 205 cells, and generated for each case cell positions similar to those measured experimentally. We fixed the positions of the cell-centers at the patch boundary. For the tested sizes of ablation (from 5 to 11 cells) and patch dimensions, the assumption of zero displacements on those external cells agreed also with the observed deformations. We measured the experimental displacements of the boundary cell centers, and the mean of their norm was in all cases below 1% of the side of the patch.

**Figure 8:**
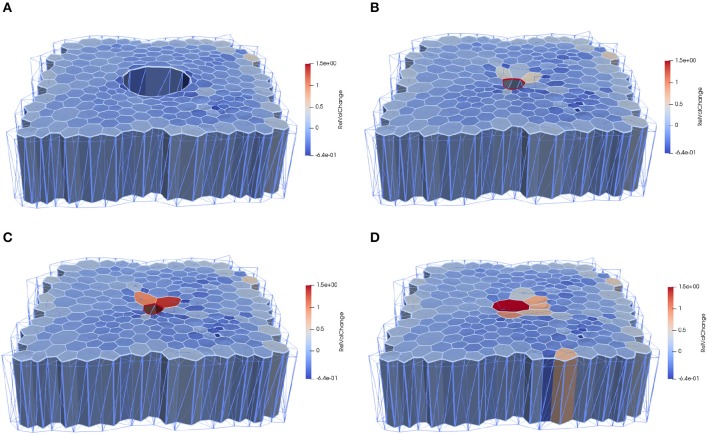
Computational epithelial tissue with 205 cells and 8 ablated cells, shown in side view for applied apical contractility of $\Upsilon_{A}^{c}=2.3$. **(A)** Apical view of epithelial computational tissue shows the maximum recoil (*t* = 10 min). **(B)** Wound edge cell before intercalation *t* = 30 min. **(C)** Wound edge cell after intercalation *t* = 32 min. **(D)** Final time step of wound closure process *t* = 52 min. The color map illustrates the relative volume change. Videos of the simulation showing the apical and basal view can be found in the Supplemental Material.

**Figure 9:**
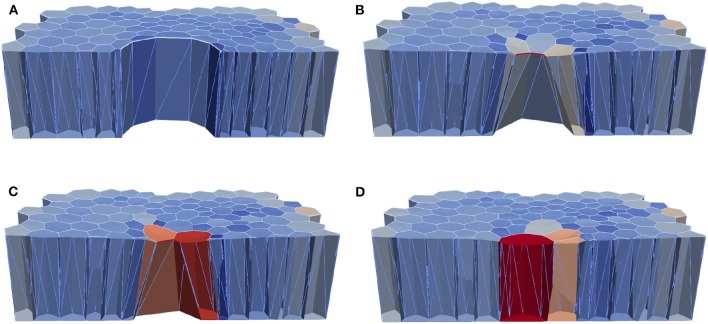
Computational epithelial tissue with 205 cells and 8 ablated cells, shown in cross-section view for applied apical contractility of $\Upsilon_{A}^{c}=2.3$. **(A)** Apical of epithelial computational tissue shows the maximum recoil *t* = 10 min. **(B)** Wound edge cell before intercalation *t* = 30 min. **(C)** Wound edge cell after intercalation *t* = 52 min. **(D)** Final time step of wound closure process. The color map illustrates the relative volume change.

**Figure 10:**
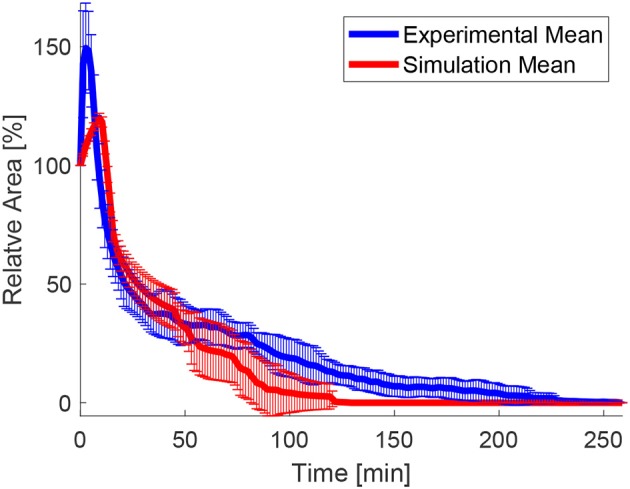
Mean values and standard deviation of the experimental and numerical area evolution.

In order to test the effects of the wound edge contractility after ablation, we measured the time evolution of the relative projected area and the relative height at the wound edge in the *in silico* model and *in vivo*. Figure 8 shows snapshots of the full simulated tissue. Two videos showing apical and basal view of the simulation can be found in the Supplemental Material (Supplementary Videos 1, 2). Figure 9 shows cross-sections through the wounded region, where unequal closure at the apical and basal sides can be observed. Figure 10 shows the standard deviation of the experimental and simulated wound area evolutions, when using the same cell center positions and similar areas for each one of the patches tested. The deviations from the mean trend are similar, but the mean values of the numerical simulations close slightly earlier than the *in vivo* wounds. More sophisticated contractility profiles were needed in order to delay the simulated closure.

**Figure 11:**
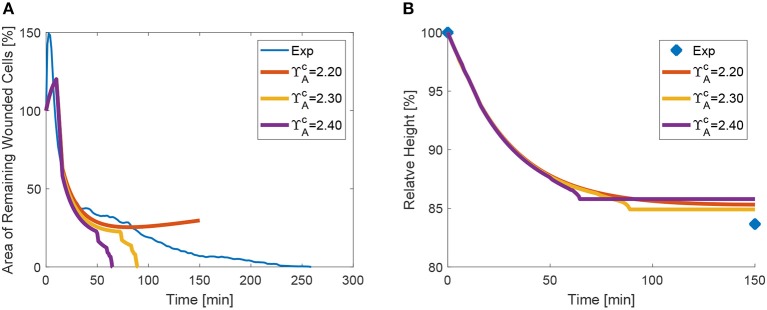
Area **(A)** and height **(B)** evolution as a function of apical purse-string contractility $\Upsilon_{A}^{c}$.

The parameter study of apical contraction $\Upsilon_{A}^{c}$ in Figure 11 shows that below a threshold value of ≈ 2.2, contractility is insufficient to close the wound, for the tested material parameters. However, the changes on the purse-string tension have a minimal effect on the relative height evolution (see Figure 11B). We remark that the material parameters also have an effect on the closure time and profile: higher values of stiffness and tissue contractility ε^*c*^ delay or may prevent closure, while higher viscosity (lower value of γ) may also delay the closure process. These material properties have been calibrated in order to match the recoil, but also the rate of closure.

**Figure 12:**
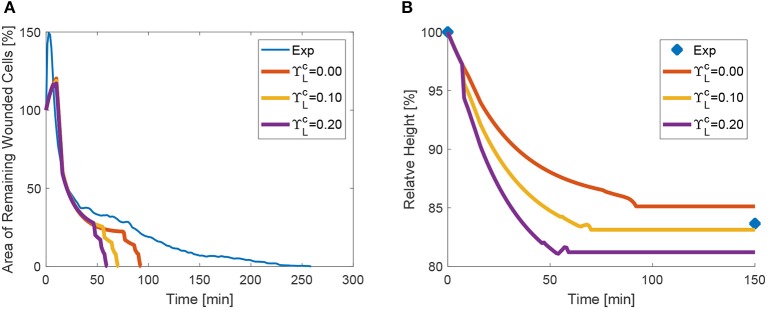
Area **(A)** and height **(B)** evolution as a function of lateral purse-string contractility $\Upsilon_{L}^{c}$.

Recently, it has been shown that changes in tissue height occur as a result of increased apicobasal contractility (Monier et al., 2015; Sui et al., 2018). We therefore also tested the effect of increased lateral contractility on wound closure. For increasing values of $\Upsilon_{L}^{c}$, the height diminishes, as expected, and the closure of the area is in turn also accelerated (see the plots in Figure 12). Despite the fact that lateral edges are inclined due to the higher area reduction in the apical side, and oppose closure, their global effect is to contribute to closure due to the volume constraint.

**Figure 13:**
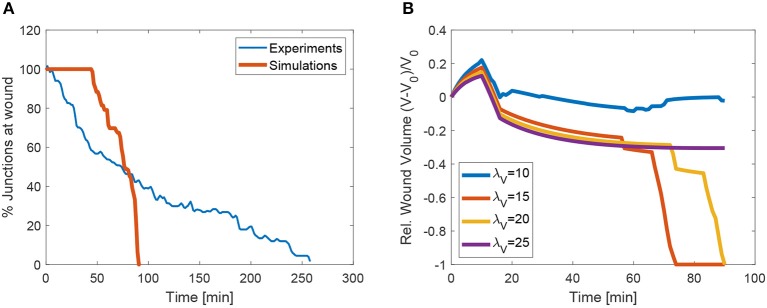
**(A)** Evolution of the mean number of junctions at apical wound perimeter *in vivo* and in the simulations. **(B)** Evolution of volume in the wounded region as a function penalization factor λ_*Vol*_. The cases with λ_1_0 and λ_2_5 did not converge.

We also analyse the evolution of the transitions, or equivalently, the number of cells at the wound edge. Figure 13A shows the evolution of the number of cells at the wound edge. While the experimental evolution is progressive, our simulations exhibit a more sudden concentration of the transitions. This may be due to the geometrical control of the transitions in the vertex discretization. We are currently investigating more accurate cell descriptions in order to obtain less drastic T1 transitions.

In order to measure the effect of the volume constraint, we checked the evolution of the apical area for different values of λ_*Vol*_. Figure 13B shows that in fact, to strict or too relaxed volume preservation may impede wound closure. In our simulations, when λ_*Vol*_ = 25 or λ_*Vol*_ = 10, the wound does not close. In the first case this is due to the need to increase the cell size to some extent in order to recover the same sized patch with fewer cells (we do not simulate cell proliferation). In the second case, when λ_*Vol*_ is too low, the recoil is too large and cell adapt to the purse-string by changing size instead of closing the wound through intercalation. In our simulations we used λ_*Vol*_ = 20, which garantees that the mean deviation of each cells remains below 10%.

## 4. Conclusions and Discussion

Recent experimental analyses have shown that wound closure is not only driven by tissue tension and contractility, but also by the rate of intercalation, so-called tissue fluidity (Tetley et al., 2019). The present model aims at extending this analysis to three dimensions, by including lateral contractility, and allowing the simulation of different intercalation at the apical and basal side.

We used the recoil process to calibrate the material parameters that characterize tissue viscoelastic properties. The rate of wound expansion just after ablation is a useful measure of tissue viscosity, which we simulated through a variable rest-length *L*(*t*) that adapts according to a remodeling rate γ. This parameter, and tissue contractility ε^*c*^ have been experimentally measured *in vitro* (Wyatt et al., 2020) for suspended monolayers. We showed that ε^*c*^ takes the value ≈ 0.3, which is close to our fitted value from the *in vivo* measurements of the area evolution. We here also showed that the recoil can be employed to evaluate this material property and that it indeed determined the final expansion.

Based on experimental observations of the evolution of myosin concentration, we applied non constant trends on the apical and lateral surfaces of the wound. We encountered a minimum value of apical tension at the wound edge, below which no closure takes place. When $\Upsilon_{A}^{c}$ is lower than approximately 2.3, the tissue in unable to surmount line tension between cells, given by ε^*c*^ in the model. It appears thus that the ratio between purse-string tension (given in the model by $\Upsilon_{A}^{c}$) and cell line tension (in the model represented by ε^*c*^) modulates the speed of closure, and that for too low values, closure may not succeed. Experimentally, too high values of line tension have been also shown to slow down or even prevent closure (Tetley et al., 2019).

We additionally observed that although lateral tension contributes in general to wound closure, depending on the tissue thickness, the net contribution of lateral purse-string contractility, regulated by $\Upsilon_{L}^{c}$, the duration of the healing process is shortened. Further inspection of the whole shape and cross-section of the wound along the apicobasal axis and accurate measurements of the height profile are necessary to corroborate this fact. This is a challenging task, given the high aspect ratio (thickness/diameter) of the cells and their high light scattering. We also note that purse-string apical tension has very minor effects on the height evolution, but lateral contractility does modify the values of tissue thickness and importantly, also area evolution (see Figure 12).

Our numerical results indicated that apical purse-string tension, when applied together with volume preservation induced a reduction of the height, due to the expansion of the tissue. We point out though that in our model, lateral contractility is applied on the whole height of the tissue. This may not be so in the real tissue, where lateral myosin may not be homogeneous along the thickness. Further discretization of the monolayer along the apicobasal axis seems necessary to simulate the specific localization of lateral myosin on the apical side. We expect that more accurate experimental measurements and model enhancements will allow us to quantify cell mechanical contribution and regulation more closely during wound closure, which as shown, are more complex than a 2D analysis may reveal.

The evolution of relative height *in vivo* and *in vitro* plotted in Figures 11B, 12B also revealed that the initial height of the monolayer was not recovered, even when the wound was fully closed. In our model, this fact can be explained by the reduced number of cells in the patch after ablation. The reduction in the total volume reduction is compensated by a height reduction, since the patch area is fixed. In the experiments, whether the patch recovers the initial height after a sufficiently long period is still under study. This analysis, and the evolution of the material properties after successive re-wounding is left for further investigations.

## Data Availability Statement

The data generated and analyzed for this study can be provided upon request to the corresponding authors.

## Author Contributions

YM conceived the project and designed the experiments. JM conceived the *in silico* model. FI, MD, and JM developed the numerical simulations. RT carried out experiments and performed experimental quantification. All authors participated in the discussions of the experiments and the modeling. JM, FI, and YM drafted the manuscript, which was completed and revised by all the authors.

## Conflict of Interest

The authors declare that the research was conducted in the absence of any commercial or financial relationships that could be construed as a potential conflict of interest.

## Supplementary Material

Supplementary Video 1 Apical view of the wound healing simulation using fitted parameters.

Supplementary Video 2 Basal view of the wound healing simulation using fitted parameters.
